# Dexamethasone Conjugates: Synthetic Approaches and Medical Prospects

**DOI:** 10.3390/biomedicines9040341

**Published:** 2021-03-27

**Authors:** Natallia V. Dubashynskaya, Anton N. Bokatyi, Yury A. Skorik

**Affiliations:** Institute of Macromolecular Compounds of the Russian Academy of Sciences, Bolshoy pr. V.O. 31, 199004 St. Petersburg, Russia; dubashinskaya@gmail.com (N.V.D.); qwezakura@yandex.ru (A.N.B.)

**Keywords:** dexamethasone, drug conjugate, drug delivery system, gene delivery system, biopolymers

## Abstract

Dexamethasone (DEX) is the most commonly prescribed glucocorticoid (GC) and has a wide spectrum of pharmacological activity. However, steroid drugs like DEX can have severe side effects on non-target organs. One strategy to reduce these side effects is to develop targeted systems with the controlled release by conjugation to polymeric carriers. This review describes the methods available for the synthesis of DEX conjugates (carbodiimide chemistry, solid-phase synthesis, reversible addition fragmentation-chain transfer [RAFT] polymerization, click reactions, and 2-iminothiolane chemistry) and perspectives for their medical application as GC drug or gene delivery systems for anti-tumor therapy. Additionally, the review focuses on the development of DEX conjugates with different physical-chemical properties as successful delivery systems in the target organs such as eye, joint, kidney, and others. Finally, polymer conjugates with improved transfection activity in which DEX is used as a vector for gene delivery in the cell nucleus have been described.

## 1. Introduction

Glucocorticoids (GC), including dexamethasone (DEX), are the most potent anti-inflammatory drugs available, but they can have quite severe acute and chronic systemic side effects [1]. The local administration of DEX in the eye or the joints, as well as inhalation and irrigation applications, can provide the correct dose of the drug directly to the inflamed site and reduce the side effects that can arise with systemic administration. However, these local methods need frequent application, which creates inconvenience for patients and may lead to patient noncompliance with the drug protocol. In addition, the use of intra-articular and intravitreal injections is limited by possible complications that can include joint infections and retinal detachment, respectively [1,2,3,4,5,6].

The administration of drugs using extended release delivery systems can control the release and increase the residence time of the drug at the target site, thereby reducing the frequency of doses and the risk of side effects [7,8,9,10,11,12]. For this reason, much research is focused on the development of DEX delivery systems with a release profile that extends from several days to months [13]. Conjugates that are able to provide prolonged release by hydrolysis of chemical bonds are of particular interest [14].

Many reviews have appeared recently on DEX delivery systems [15,16], especially those aimed at ocular [7,17] and intra-articular [8] therapies. These reviews have discussed DEX delivery using many nanocarriers, including polymer and lipid particles, dendrimers, micelles, liposomes, implants, and conjugates. However, these reviews lack a detailed description of one particularly important delivery system, namely DEX conjugates. In particular, descriptions of the methods for their synthesis and the physicochemical characteristics that affect targeted delivery are scarce. For these reasons, this review focuses on the preparation of DEX conjugates with various physicochemical properties and describes the promise these delivery systems hold for providing more appropriate sustained release profiles.

## 2. DEX as a Pharmaceutical Substance: Chemical, Pharmacological, and Biopharmaceutical Properties

**Figure 1:**
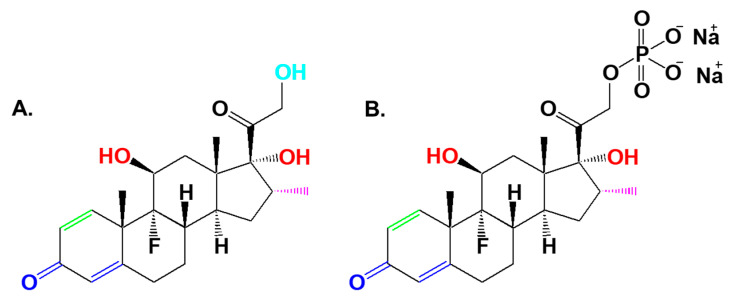
Chemical structure of dexamethasone (**A**) and dexamethasone sodium phosphate (**B**). Essential structures for pharmacological activity are highlighted in red (anti-inflammatory), in green (carbohydrate regulation), in blue (adrenocorticosteroid function), in light blue (Na^+^ retention), and in violet (Na^+^ elimination) [22,23].

DEX (9-fluoro-11β,17,21-trihydroxy-16α-methylpregna-1,4-diene-3,20-dione, Figure 1A) is a synthetic GC, a fluorinated homologue of hydrocortisone, and is included in the World Health Organization model list of essential medicines. DEX mimics the GC hormone that is secreted by the adrenal cortex mainly to regulate carbohydrate and protein metabolism. The presence of a fluorine atom in the DEX molecule makes this GC about 7 times more potent than prednisolone and about 35 times more potent than cortisone, although DEX has practically no mineralocorticoid activity. The duration of DEX action is 36–72 h, far longer than cortisone and hydrocortisone (only 5–12 h), and prednisone (12–30 h) [7,18,19,20,21].

DEX is a lipophilic substance with a solubility in water of 100 μg/mL; accordingly, DEX is soluble in acetone, ethanol, and chloroform. The water-soluble form, DEX sodium phosphate (Figure 1B), is transformed within the organism into active DEX; it has a lower lipid membrane permeability and is used as a more soluble form of the active pharmaceutical ingredient in intravenous drugs [24,25]. DEX mimics natural GCs in terms of pharmacological effects, such as anti-inflammatory, anti-allergic, immunosuppressive, anti-shock, and anti-toxicity actions [26,27].

**Figure 2:**
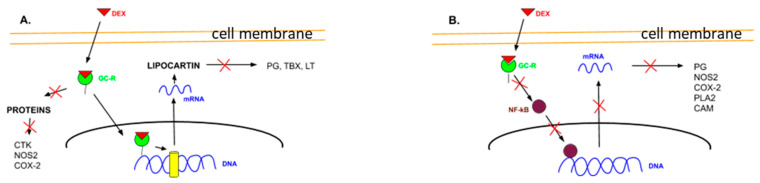
Dexamethasone action models: genomic effect (**A**) and non-genomic effect (**B**) [44].

The mechanism of DEX action can be realized in both genomic and non-genomic ways [28]. The genomic mechanism is as follows (Figure 2A): lipophilic DEX easily penetrates the cell membrane and binds to intracellular cytoplasmic GC receptors that are located in almost all organs and tissues. The resulting receptor-GC complex interacts with a specific DNA site and activates the transcription of specific genes. As a result, mRNA is synthesized and acts as a matrix for the synthesis of specific proteins that then change cell functions. For example, the synthesized protein lipocortin blocks phospholipase A2 (PLA2), thereby blocking the phospholipid destruction and the arachidonic acid formation typically carried out by PLA2. This then prevents the synthesis of inflammatory mediators (prostaglandins [PG], thromboxanes [TBX], and leukotrienes [LT]). In addition, the receptor-GC complex can suppress the production of certain proteins, such as cytokines (CTKs; including chemokines, interferons, interleukins, lymphokines, and tumor necrosis factors), as well as nitric oxide synthase 2 (NOS2) and cyclooxygenase-2 (COX-2). The genomic effects develop approximately 30 min after the formation of the receptor-GC complex.

By contrast, the non-genomic effects develop during the first minutes after the administration of high doses of DEX. These effects are realized through the interaction of the activated receptor-GC complex with nuclear factor kappa B (NF-κB) (Figure 2B). The complex blocks NF-κB and disrupts the synthesis of CTKs, including interleukin-1 (IL-1), interleukin-6 (IL-6), tumor necrosis factor alpha (TNF-α), and enzymes, including NOS2, COX-2, PLA2, and cell adhesion molecules (CAM). Through this complex modulation of cytokines, interleukins, and CAM, as well as the through the changes in the proliferation and synthesis of proteins, DEX blocks the activation of cell-mediated immunity mediated by macrophages, monocytes, basophils, fibroblasts, and lymphocytes. Thus, both anti-inflammatory and immunomodulatory effects are realized simultaneously in several ways.

In summary, the non-genomic effects of DEX include (i) stabilization of cell membranes, including mast cell membranes and organelle membranes, including lysosomes; (ii) inhibition of the activity of the mononuclear phagocyte system; (iii) suppression of leukocyte migration to the inflammation site; and (iv) reduction in the activity of endothelial cells, monocytes, macrophages, neutrophils, and fibroblasts [29,30,31,32,33,34,35,36,37,38,39,40,41,42]. The non-genomic effects of DEX are more pronounced compared to other GCs; therefore, DEX is widely used in clinical medicine, especially in the form of high-dose GC pulse therapy [27,40,43,44,45].

DEX has good oral bioavailability; it is rapidly and completely absorbed in the gastrointestinal tract and had an elimination half-life of 1–2 h. After intramuscular injection, DEX sodium phosphate is absorbed slowly, with the maximum plasma concentration reached after 7–9 h and an elimination half-life of 3–5 h. In the blood, 60–70% of the DEX binds to a specific transporter protein (transcortin). This DEX-transcortin complex is not pharmacologically active; however, bound DEX can be released and this maintains its level in the bloodstream. DEX easily penetrates the histohematological barriers, including the blood–brain barrier and placenta. It is eliminated mainly by hepatic metabolism and renal excretion. When applied topically (dermal, conjunctival, or otic), DEX is poorly absorbed and has practically no resorptive effect or associated side effects [18,19,24,46,47,48,49,50,51,52,53,54].

According to U.S. Food and Drug Administration, DEX is used in dosage forms that include tablets, solutions, and elixirs for oral administration; aerosols, ophthalmic and otic suspensions, ophthalmic ointments, and gels for topical administration; and ophthalmic inserts, intraocular suspensions, and intravitreal implants. DEX sodium phosphate is the form most often used as a solution for injection, as well as for ophthalmic and otic solutions, ophthalmic ointments, topical creams, and inhaled aerosols (including nasal applications) [55].

DEX is used to treat many conditions, including rheumatoid arthritis, bronchial asthma, systemic lupus erythematosus, autoimmune diseases, shocks of various etiologies, skin allergic diseases (neurodermatitis, eczema), and chronic rhinosinusitis, as well as to suppress transplant graft rejection [5,18,34,56,57,58,59,60,61,62,63,64]. DEX readily penetrates the conjunctiva, so it is one of the most commonly used GCs for the treatment of ocular conditions, including inflammation diseases of both the anterior (keratitis, blepharitis, allergic conjunctivitis, and dry eye) and the posterior (choroiditis, uveitis, age-related macular degeneration, diabetic macular edema, and diabetic retinopathy) segments [5,7,65,66,67,68,69,70,71].

The pharmacokinetics and pharmacodynamics parameters of DEX indicate that the development of modified DEX nanodrugs is relevant primarily for topical administration (such as ophthalmic and dermal) and other application routes (such as intravitreal, intraarticular, inhalation, nasal, and otic). The use of various nanocarriers for these types of administration would therefore focus on increasing the drug residence time at the target site through bio-adhesion, as well as on providing a controlled sustained release. In general, an ideal nanocarrier would provide a safer and more effective dosage by targeted delivery, while also reducing the drug dose, the frequency of administration, and any side effects.

## 3. Methods for the Synthesis of DEX Conjugates

Conjugates of active pharmaceutical substances with a targeting vector (for example, a polymer or a nanoparticle) are the generation 3 dosage forms that provide controlled and targeted release. As a rule, the drug molecule is linked to the vector via special linkers, and the subsequent hydrolysis provides the desired release parameters. As linkers, both fast (esters and amides) and slow (hydrazones) linkers can be used [72,73,74,75].

**Figure 3:**
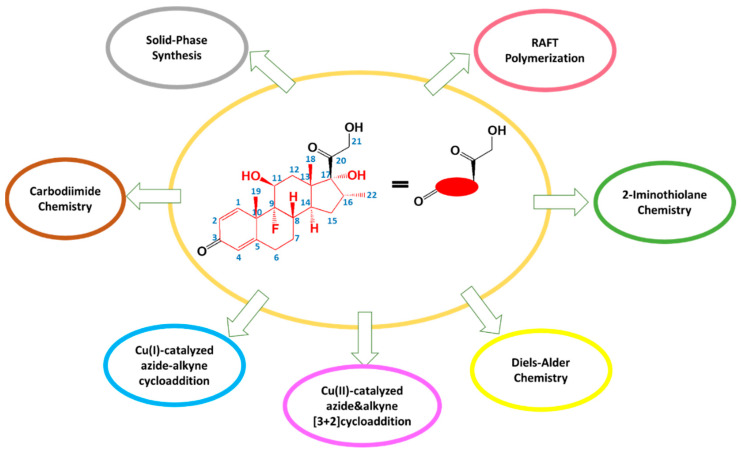
Methods for the synthesis of dexamethasone conjugates.

The 21-hydroxyl group of DEX (Figure 3) is not associated with anti-inflammatory activity and is therefore the most suitable site for conjugation [22,23]. In addition, the 21-hydroxyl is the most reactive due to its steric availability. Through this 21-hydroxyl, DEX can be covalently bound to COOH and NH_2_ groups of various vectors to form ester and amide bonds that are capable of chemical and enzymatic hydrolysis to release an active pharmaceutical substance. However, hydrolysis usually easier and faster with an ester than with an amide for release of the drug [76].

The synthesis of DEX conjugates can involve carbodiimide chemistry, solid-phase synthesis, reversible addition fragmentation-chain transfer (RAFT) polymerization, or click reactions (Cu(I)-catalyzed azide-alkyne cycloaddition, Cu(II)-catalyzed [3 + 2] azide-alkyne cycloaddition, and Diels-Alder cycloaddition), and 2-iminothiolane chemistry (Figure 3).

### 3.1. Carbodiimide Reaction

Carbodiimide chemistry uses coupling agents in the form of carbodiimides (RN=C=NR) to activate carboxyl groups as follows: (i) a COOH group reacts with the carbodiimide to produce the *O*-acylisourea; (ii) the *O*-acylisourea reacts with amines to form the needed amide. Carbodiimide chemistry is most often used to conjugate DEX with polymers that contain an NH_2_ group to form an amide (amide bond) [72] or that contain a COOH group to form an ester (ester bond) [76,77]. For attachment to the NH_2_ group of the polymer, the OH group of DEX must be functionalized with a COOH group by, for example, using anhydrides [78]. The most commonly used carbodiimides for DEX conjugation are 1-ethyl-3-(3-dimethylaminopropyl)carbodiimide (EDC), *N,N′*-dicyclohexylcarbodiimide (DCC), and *N,N′*-diisopropylcarbodiimide (DIC). Various additives, such as *N*-hydroxybenzotriazole (HOBT) or *N*-hydroxysuccinimide (NHS), are often used in carbodiimide chemistry to increase yields and decrease side reactions [72,73,79,80].

**Figure 4:**
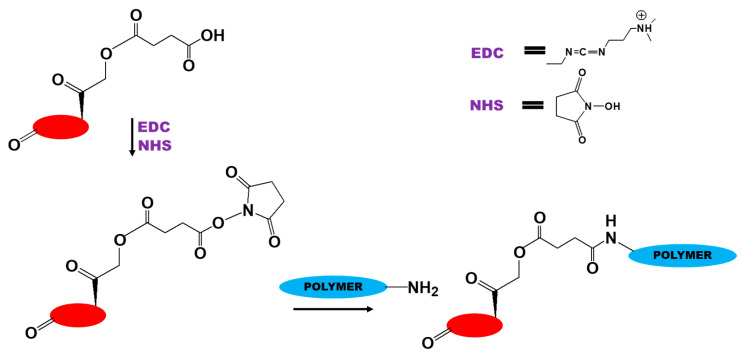
Scheme of conjugation of dexamethasone by carbodiimide chemistry.

In general, this process can be described as follows: (i) preparation of a succinimidyl ester (i.e., an activated ester) by reaction with NHS in the presence of a carbodiimide; (ii) the resulting activated ester reacts with the free amino group of the polymer to produce the corresponding amide. This method, based on an activated ester followed by the formation of an amide, is the simplest and most convenient procedure for conjugation of DEX (Figure 4) [72].

The variation involving esterification with DCC as a coupling reagent and 4-dimethylaminopyridine (DMAP) as a catalyst is named the Steglich esterification [81].

### 3.2. Solid-Phase Synthesis

**Figure 5:**
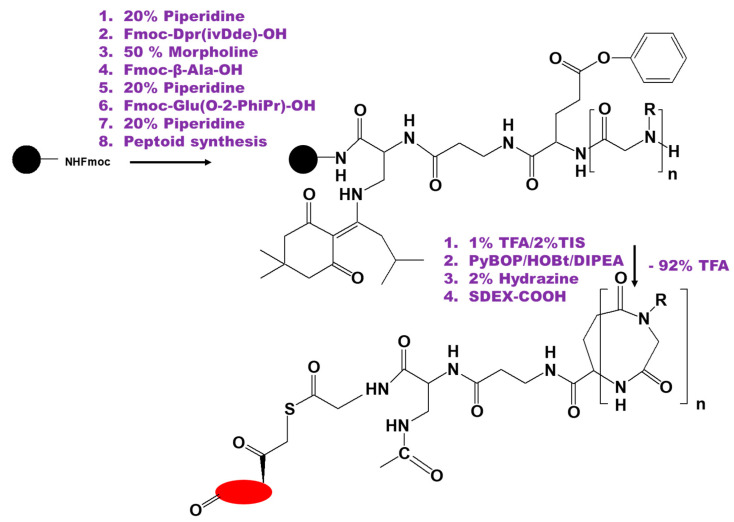
Scheme of the solid-phase synthesis of dexamethasone-peptoid conjugate [82].

Solid-phase synthesis is used to produce DEX conjugates in a step-by-step one pot reaction according to the following scheme: (i) an amino-protected amino acid binds to a solid phase material (resin) via the carbonyl group; (ii) the amino group is then deprotected and reacts with the carbonyl group of the next amino-protected amino acid (this cycle is repeated to form the desired chain); (iii) the synthesized compound is disconnected from the resin. The most widely used protecting groups for the amino groups are 9-fluorenylmethyloxycarbonyl (Fmoc) and t-butyloxycarbonyl (Boc) groups. For example, Cho et al. [82] developed the following procedure for solid-phase synthesis of cyclic and linear DEX-peptoid conjugates. The DEX-21-thiopropionic acid (SDex-COOH) (with high affinity for the GC receptor ligand-binding domain, and compatibility with the solid-phase synthesis) was first synthesized [83] Cyclic DEX-peptoid conjugates were formed by attaching (Fmoc)-di-aminopropionic acid (1-(4,4-dimethyl-2,6-dioxocyclohex-1-ylidene)-3-methylbutyl)-OH onto the Rink Amide AM resin, where one amino group was used for the synthesis of the peptoid sequences and the other for the attachment of the DEX derivative (Figure 5). Benzotriazole-1-yl-oxy-tris-pyrrolidino-phosphonium hexafluoro-phosphonate (PyBOP) was used as a linker between the DEX moiety and the peptoids. The desired cyclic DEX-peptoid conjugates were formed by the deprotection of 1-(4,4-dimethyl-2,6-dioxocyclohex-1-ylidene)-3-methylbutyl with 2% hydrazine and coupling with SDex-COOH. The resulting conjugates were cleaved from the resins with 92% trifluoroacetic acid (TFA) containing 3% triisopropyl-silane and 5% water, and then purified by reverse-phase high performance liquid chromatography.

### 3.3. RAFT Polymerization

DEX conjugates can also be obtained by reversible addition-fragmentation chain transfer polymerization or RAFT polymerization. Typically, a RAFT polymerization system consists of (i) a monomer; (ii) an initiator (a radical source, also can control molecular weight (MW) and polydispersity); and (iii) a RAFT agent (a chain transfer agent). The monomers for the synthesis of RAFT conjugates of DEX can include (hydroxypropyl)methacrylamide (HPMA) and DEX-containing monomers. Radical initiators, such as azobisisobutyronitrile (AIBN) and RAFT agents in the form of thiocarbonylthio compounds, such as S,S′-bis(α,α′-dimethyl-α′′-acetic acid)-trithiocarbonate (CAT), are most often used for producing DEX-containing RAFT polymers. RAFT systems usually conjugate a DEX molecule at position 3C; as a rule, these conjugates exhibit sustained release (zero order kinetics) for several months and are therefore used for targeted intra-articular delivery.

**Figure 6:**
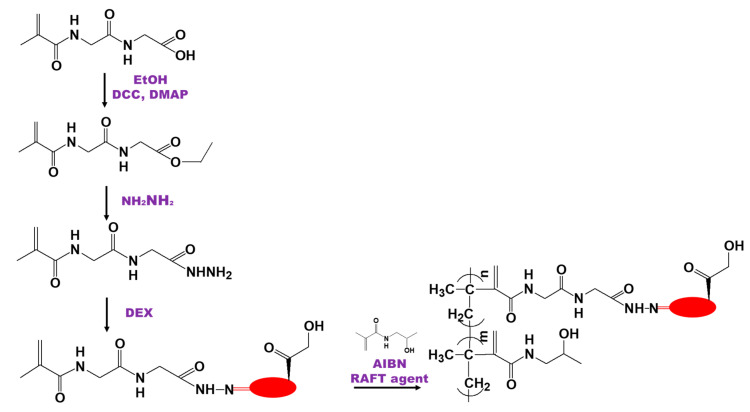
Scheme of the synthesis of dexamethasone-(hydroxypropyl)methacrylamide conjugate by reversible addition fragmentation-chain transfer polymerization [84].

Liu et al. [84] developed a pH-sensitive DEX containing an HPMA copolymer conjugate for the improved treatment of rheumatoid arthritis. A DEX-containing monomer was first synthesized and then copolymerized with HPMA using RAFT polymerization. The obtained conjugates had MW of 34,000 and a DEX content of 100 mg/g. The developed synthesis scheme included the following steps: (i) synthesis of *N*-methacryloyl glycyl glycyl hydrazide (MA–Gly–Gly–NHNH_2_), as follow: to *N*-methacryloyl glycylglycine (MA–Gly–Gly–OH) was added small amount of inhibitor (tert-octyl pyrocatechine) to prevent polymerization and DCC as the coupling agent. The reaction mixture was stirred for 2 h at 0 °C and 2 h at room temperature and then filtered to remove dicyclohexylurea at 0 °C. Hydrazine hydrate was then added into the filtrate and the solution was stirred for 4 h at room temperature, the product was precipitated with hexane and washed with ethanol-hexane; (ii) synthesis of pH-sensitive MA–Gly–Gly–NHN=DEX was as follows: MA–Gly–Gly–NHNH_2_ and DEX were dissolved in methanol, and acetic acid was added as a catalyst. The solution was purged with Argon, stirred for 3 days at room temperature, the solvent was evaporated, and the product was purified by flash column chromatography; (iii) the synthesis of the DEX-HPMA conjugate via RAFT co-polymerization was as follows: HPMA and MA–Gly–Gly–NHN=DEX were dissolved in methanol/DMF with AIBN as an initiator and CAT as the RAFT agent (Figure 6).

The in vitro study showed no DEX release at pH 7.4, but a linear release (a zero-order release) of DEX at pH 5.0 at a rate of about 1% of the loaded drug per day (~14% in 14 days). The in vivo study (endpoint bone mineral density test and histology grading) showed strong and long-lasting anti-inflammatory and joint protection effects.

### 3.4. Click Reactions

Click chemistry is a product synthesis procedure that combines small modular units and is characterized by “green” one-pot synthesis conditions, high specificity, and high product yield, as well as minimal and non-toxic by-products [85]. Therefore, click reactions, including Cu(I)-catalyzed azide-alkyne cycloaddition and Cu(II)-catalyzed [3 + 2] azide-alkyne cycloaddition, have been used for the synthesis of DEX conjugates [86,87,88,89,90].

**Figure 7:**
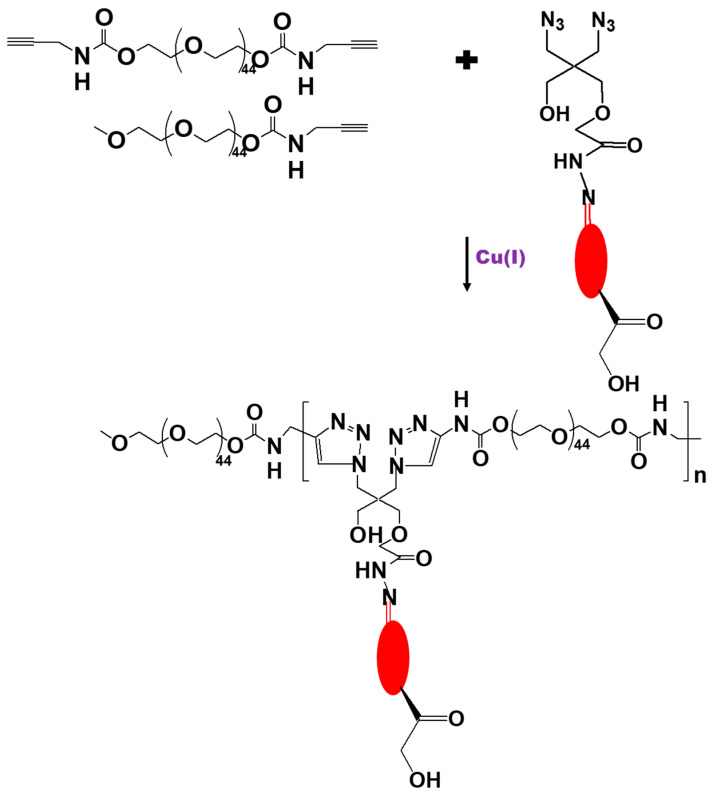
Scheme for the synthesis of a dexamethasone conjugate with acetylene polyethylene glycol by Cu(I)-catalyzed 1,3-dipolar cycloaddition.

Liu et al. [86] synthesized a DEX–polyethylene glycol (PEG) conjugate (DEX-PEG) using Cu(I)-catalyzed 1,3-dipolar cycloaddition that included an acid-labile hydrazone bond for DEX release in a pathophysiological environment. The first product, 2,2-bis(azidomethyl)propane-1,3-diol, was synthesized and then monocarboxylated with methyl 2-bromoacetate. The carboxylate was then converted to hydrazide by reacting with excess hydrazine. As the final step, the acid-labile hydrazone bond was formed by coupling DEX with the hydrazide compound (Figure 7). Using click copolymerization, the DEX-PEG was synthesized by mixing the DEX-containing monomer, acetylene PEG, acetylene monomethyl ether PEG (mPEG) (as a chain terminator), tris-(hydroxypropyl triazolyl methyl)amine (as a stabilizing agent), and the Cu(I) catalyst. In vitro release studies showed a linear DEX release profile at pH 5.0 (approximately 10% of DEX was released in 17 days). By contrast, less than 1% of the DEX was released at pH 6.0, and no DEX release was detected at pH 7.4 after 17 days. An in vivo study comparing the conjugate to the equivalent dose of free DEX showed that a single administration of the DEX-PEG conjugate provided a long-term (>15 days) reduction in ankle joint inflammation in a rat model of adjuvant-induced arthritis.

**Figure 8:**
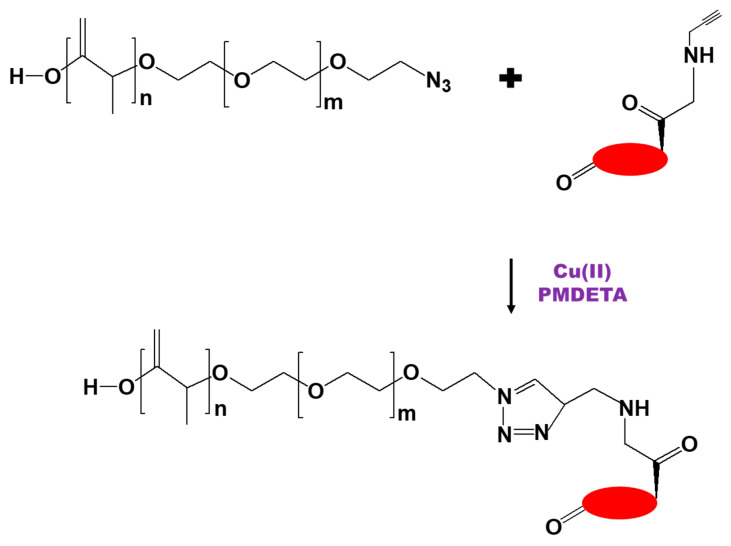
Scheme for the synthesis of a dexamethasone conjugate with N_3_-polyethylene glycol-polylactic acid copolymer by Cu(II)-catalyzed cycloaddition [87].

Karandish et al. [87] used Cu(II)-catalyzed azide-alkyne cycloaddition to design nucleus-targeted, stimuli-responsive polymersomes for the delivery of anticancer drugs to the nuclei of pancreatic cancer cells. In this case, the DEX group was used to improve the transport of the drug carriers to the nuclei via dilation of the nuclear pore. An alkyne-DEX derivative was first synthesized under N_2_ using methanesulfonyl chloride and propargylamine; this derivative was then conjugated to a N_3_-PEG-polylactic acid copolymer or a PEG-*S-S*-polylactic acid copolymer by the Cu(II) complex (a mixture of CuSO_4_ and pentamethyl diethylenetriamine [PMDETA]) catalyzed cycloaddition reaction) (Figure 8). The nuclear uptake studies showed that the targeted DEX-containing polymersomes were present in pancreatic cancer cell nuclei after 3 h of incubation, while non-targeted DEX free polymersomes did not penetrate the cells.

### 3.5. Diels–Alder Reaction

**Figure 9:**
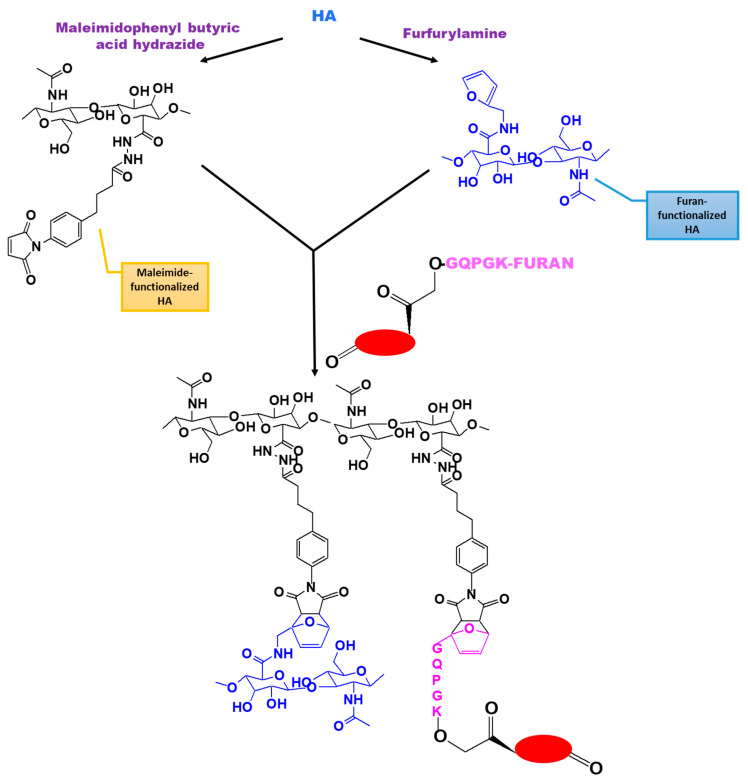
Scheme for the synthesis of a dexamethasone conjugate with hyaluronic acid maleimide by Diels–Alder cycloaddition [91].

The Diels–Alder [4 + 2] cycloaddition, which is a chemical reaction between a conjugated diene and a substituted alkene (dienophile) that forms a substituted cyclohexene derivative, can also be used for the design of DEX conjugates. For example, Jia et al. [91] developed the DEX conjugates as an adipogenic factor with a magnetic hyaluronic acid (HA) (MW 1,600,000) nanosphere system for adipose tissue engineering. The magnetic HA nanosphere system was prepared between the furan and maleimide groups of HA derivatives (conjugation of furfurylamine to the carboxylic group on HA, with amide bond formation) by aqueous Diels–Alder cycloaddition. The furan-functionalized DEX-peptide (DEX-GQPGK) was then synthesized via solid -phase synthesis using a synthetic Fmoc-protected amino acid analog. The 3-furoic functionality was directly incorporated during the solid phase synthesis, and the DEX-NHS ester was introduced by reaction with the *N*-terminal primary amine. Finally, a HA nanosphere system was prepared by conjugating HA-furan and DEX-GQPGK-furan with HA-maleimide via the Diels–Alder cycloaddition (Figure 9). The resulting particles had a size of about 150 nm and provided a sustained release of DEX (13% in 24 h, PBS, pH 7.4). In vitro cytotoxicity tests demonstrated that incorporation of DEX into HA nanospheres gave a product that showed high efficiency in promoting human adipose-derived stem cell viability.

### 3.6. Traut’s Reaction

**Figure 10:**
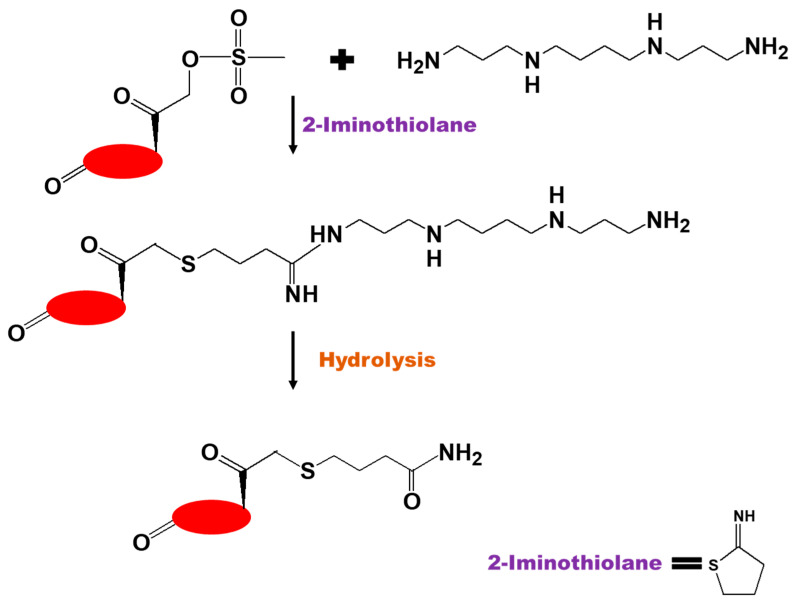
Scheme for the synthesis of dexamethasone -NH_2_ by 2-iminothiolane (Traut’s reagent) chemistry [22].

2-Iminothiolane (Traut’s reagent) chemistry. Gruneich et al. [22] developed a procedure for the introduction of a NH_2_ group into DEX to create a cationic DEX for gene delivery and anti-inflammatory activity. This was achieved by a 45 min one-pot reaction between the aliphatic polyamine spermine and DEX-21-mesylate (DEX-21-methanesulfonate) in the presence of 2-iminothiolane according to the following mechanism. Traut’s reagent was selectively ring-opened by the primary amines on spermine to form a hydrolytically sensitive amidine bond between spermine and iminothiolane and a reactive thiolate anion that reacted with the α-keto mesylate on the 21 position of DEX-mesylate to form α-keto thioether between spermine and iminothiolane. The resulting conjugate was hydrolyzed in 1 M NaOH for 20 min, resulting in the disintegration of the amidine linkage between spermine and iminothiolane and the formation of a DEX-amide consisting of a 21-substituted butyl thioether amide side chain on DEX (Figure 10).

This procedure has two benefits. One is that the amino-functionalized DEX can be used for conjugation with carriers containing COOH groups. The second is that Traut’s reagent can be used to conjugate DEX to amine-containing carriers. This method is most often used for the synthesis of gene delivery systems [92,93,94,95,96,97,98,99].

## 4. Dexamethasone Conjugates as Delivery Systems with Modified Release

DEX is widely used to synthesize modified-release conjugates without compromising pharmacological activity. For example, Zacchigna et al. [100,101] conjugated DEX and DEX/theophylline to PEG (MW 6000 and 10,000). The addition of DEX to a polymer aminated by a 2-amino-1,3-propanol (by method [102]) was carried out via a succinyl linker using carbodiimide chemistry (PEG-NH_2_ + Succinyl-DEX). The in vitro DEX release was pH dependent and amounted to about 5.5% over 24 h in phosphate buffered saline (PBS) (pH 7.4), less than 1.8% over 6 h in artificial gastric juice (pH 1.5), and more than 45% over 24 h in simulated duodenal fluids (pH 6.8). An in vivo study on a rat model showed that the bioavailability of conjugated DEX after oral administration was about 1.5 times higher than that obtained for free DEX.

Keely et al. [103] conjugated DEX to the amino group of poly(dimethylamino)ethyl methacrylate via bromoacetic anhydride and a quaternization reaction. The amount of conjugated DEX was 9 and 18 mol per 1 mol of polymer. The resulting conjugates showed equivalent bioadhesion to Caco-2 cells and mucoadhesion to E12 cells to that of the starting polymer and had anti-inflammatory activity comparable to that of pure DEX.

Choksi et al. [104] obtained a DEX-prodrug based on a poly(amidoamine) generation 4 (PAMAMG4) dendrimer. The DEX-PAMAMG4 conjugates were prepared by the EDC-coupling reaction using succinic anhydride as the spacer to reduce steric hindrance on the surface of the dendrimer. Conjugation of succinic anhydride to the NH_2_ group of PAMAMG4 resulted in the formation of a monocarboxylic acid conjugate, which was then conjugated with the hydroxyl group of DEX. The second synthesis option included the initial synthesis of succinyl-DEX, which introduced a carboxyl group into the DEX molecule; that group was then conjugated to the NH_2_ group of the PAMAMG4 dendrimer. The amount of conjugated DEX was 64.19 μg/mg for the conjugate of succinic-PAMAMG4 with DEX and 32.37 μg/mg for the conjugate of PAMAMG4 with succinyl-DEX (due steric hindrance). The developed DEX conjugates inhibited TNF-α approximately 1.5 times more efficiently than liposomes containing the same amount of DEX.

Wang et al. [105] synthesized of DEX-cytokinin ligands with a spacer linkage. DEX was attached to the purine ring of 6-benzylaminopurine at positions 2, 8, or 9. To achieve this, DEX was modified by periodate oxidation to convert the hydroxymethyl ketone moiety into a carboxyl group for use in conjugation to the spacer by amide formation (NHS/DCC chemistry).

Numpilai et al. [75] conjugated DEX with aldehyde-functionalized porous silica materials via a pH-responsive hydrazone bond (a linker of adipic acid dihydrazide) by the reaction of the hydrazide groups with carbonyl groups of DEX. The loading efficiency was 15–85 μg/mg. In vivo experiments showed a pH-dependent sustained release of DEX of 12–37% for 10 days at pH 4.5 and less than 1.5% at pH 7.4.

These examples show that various vectors and platforms can be used to immobilize DEX and that these systems can be successfully used for targeted drug delivery to various tissues and organs.

### 4.1. Ocular Delivery Systems

Topical steroid administration in the form of eye solutions, suspensions, or ointments is less likely to cause serious side effects compared with systemic use (injection, oral), and it is more comfortable for the patient. However, topical application has low ocular bioavailability due to the rapid precorneal clearance and poor corneal permeability [106,107,108,109]. DEX conjugates with various bioadhesive polymers can increase the precorneal residence time and enhance corneal permeability, thereby increasing ocular bioavailability.

The treatment of retinal diseases (diabetic macular edema, and diabetic retinopathy) requires the development of intravitreal forms that do not need surgical implantation [110,111]. Yu et al. [106] developed a DEX-peptide conjugate for topical ocular administration by forming a biodegradable ester linker of succinic anhydride that could spontaneously self-assemble into micelles in an aqueous solution. The conjugates were obtained by a classic solid-phase peptide synthesis method using 2-chlorotrityl chloride resin and *N*-Fmoc-protected amino acids (*N*-protected amino acids). Succinyl-DEX was coupled to the peptide using *N,N*-diisopropylethylamine and *O*-benzotriazol-1-yl-tetramethyluronium hexafluorophosphate as the coupling agent. The formed micelles were 150 nm in size and carried a negative charge of −18 mV due to the ionized carboxyl groups of the amino acid residues on the micelle surface. An in vitro release study (PBS containing 20 U/mL esterase, pH = 7.4) showed that almost a 100% release of DEX in 48 h. The in vitro anti-inflammatory efficacy of the DEX-conjugates was monitored by the secretion of NO and pro-inflammatory cytokines, such as IL-6 and TNF-α, and the secretion was almost identical to that of the native form of DEX. An in vivo test in rabbit eyes showed a slight inflammatory response with minimal inflammatory exudate in the anterior chamber at 24 h after application of the DEX-conjugates, whereas the control group exhibited a severe inflammatory cell response.

Yavuz et al. [76] fabricated subconjunctival and intravitreal DEX-poly(amidoamine) (PAMAM) dendrimer conjugates for retinal delivery. The conjugates were synthesized by esterification of a PAMAM-carboxyl group with a DEX-hydroxyl group in the presence of DCC and DMAP. The resulting conjugates had substitution degrees of 4 and 10, sizes of 130–185 nm, and ζ-potentials of −17 to −57 mV. A hydrolysis study in the presence of corneal and sclera-choroid-retinal pigment epithelium tissues showed that enzymatic degradation of the conjugates was very slow, as less than 8% of the DEX was released in 6 days. Fluorotron analysis indicated low conjugate levels in the vitreous and retina following subconjunctival injection but high levels following intravitreal injection of the conjugate (1 mg/mL). Furthermore, the conjugate level was higher in the retina-choroid conjugate level than in the vitreous, indicating that the DEX-conjugate had penetrated to the retina from the injection site.

Wang et al. [81,112] developed conjugates of porous silicon dioxide microparticles for the intravitreal controlled release of DEX. For this, amine-functionalized porous silica particles were reacted with succinic anhydride to obtain a carboxylic acid functionalized surface (the carboxylic acid group resulted from ring opening of the succinic anhydride through a reaction with the amine group on the surface of the particles). DEX was then conjugated via a Steglich Esterification Reaction between the hydroxyl of DEX and that carboxyl group (in the presence of DCC and DMAP). The drug loading efficiency was between 6 and 10%. An in vitro drug release study revealed that DEX release from conjugates (in medium of cell culture grade water) was sustainable for over 90 days; this was 80 days longer than the release from free DEX or from infiltration-loaded porous silica particles in the same setting. In addition, a pilot in vivo study in a rabbit eye model demonstrated that a single intravitreal injection (3 mg) gave a free drug level at 2 weeks of 110 ng/mL, which is significantly higher than the therapeutic level.

### 4.2. Intra-Articular Delivery Systems

Local intra-articular injections are characterized by rapid excretion from the joint space and slow diffusion of drugs through a dense avascular cartilage matrix consisting of negatively charged glycosaminoglycans. Targeting GC-conjugate delivery to joints increases the drug residence time in the joint, improves the therapeutic effectiveness, and decreases the systemic toxic reactions in healthy tissues, thereby enhancing the safety of prolonged use of drugs such as DEX [78].

Sangar et al. [72] used a cystine-dense peptide (CDP) capable of rapidly accumulating in cartilage for the development of conjugates with DEX. CDPs are mini-proteins (typically 20 to 60 amino acids in size) with a rigid three-dimensional structure that contains least three disulfide bonds (cystines). DEX conjugates with CDP were obtained by carbodiimide synthesis using cysteine (Boc-cysteine) as a stable linker or dimethyladipic acid as a labile linker. The conjugates were obtained in two stages: first, DEX-linker-NHS esters were synthesized in the presence of EDC. The obtained compounds were then conjugated with CDP in the presence of *N*-methylmorpholine. An in vivo study of the biodistribution of the conjugates in rats, mice, and human cartilage explants showed that their content in the knee joint was significantly higher (50–100 times) than in blood, muscle, and liver. The administration of a stable linker-based conjugate also did not lead to the detection of free DEX in the knee cartilage. The labile ester linker-based conjugates remained primarily intact in circulation and released the drug in the joints (the hydrolysis half-life was 9.9 and 22.4 h in rats and humans, respectively). In addition, the conjugated DEX showed pharmacokinetic parameters, such as C_max_ (71 ng/mL in 6 h) and AUC (700 h·ng/mL), that were significantly different from those of the same dose of DEX phosphate (C_max_ = 224 ng/mL in 30 min and AUC = 1060 h·ng/mL). These data confirmed that the DEX-CDP conjugates have a targeted bio-distribution and they decrease the systemic effect of DEX.

He et al. [78] fabricated DEX-avidin constructs to improve intra-articular delivery. Avidin, a tetrameric cationic glycoprotein, was used as a vector for targeting cartilage. Avidin has a size of less than 10 nm and a charge between +6 and +20mV. It is able to weakly and reversibly bind to anionic glycosaminoglycans; therefore, it can penetrate through the full thickness of cartilage. In addition, succinic, dimethylglutaric, and phthalic anhydrides were used to form hydrolyzable ester linkers. These nano-constructions were obtained in three stages. First, a PEG (MW 10,000)-NHS-biotin complex (with a degree of biotinylation of 1.15) was synthesized, and then succinyl-DEX, dimethylglutaryl-DEX, or phthalyl-DEX were added using EDC/NHS chemistry. The three resulting structures were loaded with avidin by exploiting its high affinity for biotin. The resulting succinyl, dimethylglutaryl, and phthalyl conjugates had 6.6, 1.6, and 3.3 DEX molecules per 1 conjugate molecule, and the drug loading content was 15.7, 3.8, and 7.8%, respectively. In this case, the length of the carbon spacer (succinyl, dimethylglutaryl, or phthalyl) also influenced the DEX release profile and the half-life of the ether bond. About 70% of the loaded DEX was released during the first 24 h from the succinyl derivative, with a half-life of 6.8 h. By contrast, the dimethylglutaryl and phthalyl derivatives each released, approximately, 80–90% of the DEX in 360 h, with half-life values of 79 and 86 h, respectively (into both PBS and synovial fluid). In cartilage explant culture models of osteoarthritis, a single 10 μM low dose of DEX-conjugates effectively suppressed IL-1α-induced glycosaminoglycans loss, cell death, and the inflammatory response significantly better than free DEX over 2 weeks.

Bajpayee et al. [73] used EDC/NHS chemistry to covalently conjugate DEX with biotinylated-PEG(MW 2300)-avidin via fast (ester) and slow (hydrazone) pH-sensitive release linkers. Non-covalent analogs of DEX with avidin/PEGylated avidin were also prepared to compare the release profiles. The DEX release from non-covalent compounds (PBS, pH 7.4) reached ~70% in 3 h. By contrast, conjugation of DEX to avidin via an ester linkage dramatically slowed the release at pH 7.4, with a mean lifetime of 21 h. Conjugation of DEX to avidin via a hydrazone linkage caused a greater slowing of DEX release (the maximum release was 30% in 350 h at pH 7.4). However, in an acidic environment at pH 4.0, the DEX release was 80% in 150 h, with a mean half-life of 60 h. In vitro tests of the biological activity of these conjugates using a model of cartilage catabolic injury showed that a single dose of DEX-avidin conjugates eliminated IL-1α-induced cell death and suppressed cytokine-induced glycosaminoglycan loss over 3 weeks.

Formica et al. [113] obtained a DEX-chitosan (MW 100,000–200,000; deacetylation degree of 88%) conjugate and a DEX-collagen type II-binding peptide conjugate. Both polymeric vectors promoted deep and sustained infiltration of the DEX into full-thickness cartilage via the strong electrostatic interactions of chitosan with the negatively charged cartilage extracellular matrix or via special interactions with cartilage-specific collagen type II bundles. The chitosan conjugate was prepared in a two-step reaction. DEX was first methacrylated on its primary alcohol, thereby introducing a hydrolysable ester bond. The DEX methacrylate was then conjugated to the primary amine of chitosan with a Fe(III) catalyst. The collagen type II binding peptides were conjugated with DEX via an orthogonal glutamic acid residue by Steglich esterification, and glycine was added as a steric linker. In both cases, active DEX was released from the conjugates by ester linkage hydrolysis. Thus, conjugation of DEX with cartilage-affine carriers increased its binding and therapeutic efficacy when compared to the free drug. Both DEX conjugates significantly reduced the levels of inflammatory markers (IL-6, COX-2, and MMP-13) and slowed the loss of glycosaminoglycans in an ex vivo model.

Wei et al. [114] synthesized DEX conjugates with the HPMA copolymer for implantation in joints. The DEX-HPMA conjugates were synthesized by a RAFT copolymerization of HPMA (MW 20,000; 30,000; and 40,000), MA-Gly-Gly-NHN=DEX, and *N*-methacryloyl tyrosine amide (MA-Tyr-NH_2_). The amount of conjugated DEX increased with an increase in MW from 280 to 350 mol%. The pharmacokinetic parameters of the tested conjugates were also dependent on MW, as an increase in MW from 20,000 to 40,000 led to a ~26-fold increase in the AUC, a ~26-fold decrease in the total clearance, and an increase in the half-life associated with the elimination phase from 27 to 36 h.

Quan et al. [115] also synthesized a DEX-HPMA conjugate by RAFT copolymerization. The DEX content in the copolymer was 92 mg/g. An in vivo testing in the mouse model of collagen-induced arthritis showed that one injection of DEX-conjugate inhibited the clinical signs of disease for 1 month, which was comparable to the inhibitory effect of an equivalent daily dose of free DEX.

Ren et al. [116] designed a DEX-HPMA conjugate by RAFT copolymerization to prevent osteolysis. The DEX content was 140 mg/g. An in vivo study showed that monthly DEX conjugate administration in a dose equivalent to free DEX was as effective as free DEX but did not cause systemic side effects or systemic bone loss (a major adverse side effect of GC therapies).

Quan et al. [117] designed a DEX-HPMA conjugate (MW 14,000; 24,000; and 42,000) using RAFT polymerization and obtained a conjugate with a DEX content of 118 mg/g. The resulting conjugate was stable in rodent plasma: less than 0.4% of the DEX was released in 72 h. An in vivo study in the adjuvant-induced arthritis rat model showed that the MW of the conjugates influenced the pharmacokinetics and biodistribution parameters of DEX. The half-life values were 31, 17–18, and 13 h for conjugates based on polymers with MW of 42,000, 24,000, and 14,000, respectively. The increase in MW also enhanced the arthrotropism of the conjugates. Thus, the conjugate with highest MW of 42,000 was most selectively accumulated in ankle synovial tissue.

Wang et al. [118] developed a pH-sensitive DEX drug delivery system based on a HPMA copolymer. The MW of the conjugate was 73,000 and the amount of conjugated DEX was 50 mg/g. An in vitro study showed a pH-dependent, zero-order release profile for 14 days. Overall, 14% of the DEX was released at pH 5.0, while the release was only 2 and 1% at pH 6 and pH 7.4, respectively.

Bae et al. [80] produced conjugates of DEX with HA (MW 1,500,000) for inclusion in PEG (MW 4000)-bis-(2-acryloyloxy propanoate) scaffolds for point-of-care cell therapy for orthopedic repair. A DEX-HA acid conjugate was synthesized via a succinyl linker using a carbodiimide active ester-mediated coupling reaction. Succinic anhydride was first coupled to the hydroxyl group of DEX, and then DEX-monosuccinate was coupled to the hydroxyl groups of HA in the presence of carbonyldiimidazole and triethylamine. The amount of conjugated DEX was 3.01% (*w/w*). The DEX release into enzyme-containing PBS was approximately 80% over 12 days and reached a total of 90% over 24 days. In PBS alone, approximately 10% of the DEX was released in 24 days.

### 4.3. Kidney Delivery Systems

Hu et al. [119] developed E-selectin-targeted sialic acid-PEG-DEX micelles for improving anti-inflammatory efficacy for the treatment of acute kidney injury. Sialic acid was used as a vector for targeting E-selectin, which is expressed on inflammation-activated vascular endothelial cells. The synthesis of conjugates was accomplished in two steps: an esterification reaction was first conducted between the carboxyl groups of PEG bis(carboxymethyl) ether (HOOC-PEG-COOH) and the hydroxyl group of DEX in the presence of DCC and DMAP; next, sialic acid was conjugated via the esterification reaction between carboxyl group of PEG-DEX and the hydroxy group of sialic acid. The conjugated DEX content in the conjugates was 15.9%. The obtained conjugates self-assembled into micelles in an aqueous medium additionally loaded with DEX. The resulting particles were 23–45 nm in size, the ζ-potential was −7 to −13 mV, and the loading efficiency was 6–10%. In this case, the conjugates released 40% of the DEX in 50 h, while the micelles had a two-phase release: a quick release of 70% in 10 h, followed by a release of 85% of the DEX in 50 h. The conjugated DEX content in in vivo studies showed that the obtained DEX nanocarriers selectively accumulated in the kidneys of the mouse model of acute kidney injury, where they significantly reduced the levels of cytokines (TNF-α and IL-6), as well as markers of oxidative stress (superoxide dismutase, malondialdehyde, and myeloperoxidase), compared to free DEX for 7 days.

Yuan et al. [120] prepared a HPMA copolymer-based DEX pro-drug for the treatment of lupus nephritis. The conjugates were synthesized by RAFT copolymerization [84]. An in vivo study on the (NZB × NZW)F1 and NZW mouse models showed that monthly DEX-conjugate injections completely abolished albuminuria and decreased macrophage infiltration by about 3-fold compared with daily free DEX treatment. In addition, the DEX-conjugate treatment did not lead to significant deterioration of bone quality, in contrast to free DEX treatment.

Ren et al. [121] synthesized an acid-labile HPMA copolymer-DEX conjugate to prevent inflammation and bone damage in the calvaria osteolysis model by method [84]. An in vivo study on the mouse calvarial osteolysis model demonstrated an osteoprotective action of developed conjugates.

### 4.4. Antitumor Delivery Systems

The tumor microenvironment is abnormal and heterogeneous. In one process (the so -called Warburg effect), cancer cells use glucose inefficiently and produce large amounts of lactic acid, which acidifies the tumor tissue. In addition, the enhanced permeability and retention effect (EPR effect) shows that tumors have abnormal blood vessels and ineffective lymphatic drainage, which allows large molecules and particles (<500 nm) to penetrate into the tumor tissues and remain in them for a long time. The Warburg and EPR effects are the rationale for pH-sensitive drug delivery systems that can deliver and release drugs principally in tumor tissues [122,123,124,125,126]. Therefore, the development focuses on targeted delivery conjugates that are sensitive to specific factors of the tumor medium (high glutathione concentration, and pH~5–6). When administered intravenously, these systems selectively accumulate in the tumor, driven by the EPR-effect, and then the drug is released in response to the low pH and redox environment.

DEX is a useful molecule for nuclear-targeted drug delivery. In the cytosol, DEX binds to GC receptors and eventually translocates to the nucleus, increasing the nuclear pores size to almost 100 nm, thereby facilitating nuclear delivery of the drug carrier [127,128,129,130,131]. A second useful factor is the anti-inflammatory effect of DEX. Chronic inflammation is an important oncogenic factor; for example, tumor-associated macrophages promote tumor cell hyperplasia via the secretion of growth factors, promote angiogenesis via the secretion of vascular endothelial growth factor (VEGF), promote metastasis and invasion tumors via secretion of matrix metalloproteinases, and also suppress adaptive immunity via secretion of various immunosuppressive cytokines [77,132,133,134,135,136].

Tran Thi et al. [127] synthesized a reduction-responsive methoxy-PEG (MPEG)-DEX conjugate as an intracellular targeted drug delivery carrier. In this case, DEX molecules were used to target the nucleus of the tumor cell. The conjugates were synthesized via multistep synthesis; the degree of substitution of DEX was 1.4–1.6. The terminal hydroxyl group of the MPEG was first activated by p-nitrophenyl chloroformate. The disulfide linker cystamine or a control linker ethylenediamine were then connected, and DEX was coupled using carbodiimide chemistry. The amphiphilic DEX-conjugates self-assembled in aqueous solutions to form nanoparticles with a size range of 130 to 150 nm. Both conjugates maintained good colloidal stability in bovine serum albumin for more than 1 week; however, in the presence of 10 mM dithiothreitol (a model thiol-containing stimulus) containing disulfide bonds, the nanoparticles were completely destroyed in 3 h. These results showed that DEX conjugates containing a disulfide linker could maintain their colloidal stability in systemic circulation and then disintegrate in the tumor cell environment, thereby increasing the intracellular release of the drug.

Ma et al. [77] synthesized a redox and pH dual sensitive DEX conjugate with MPEG-block-poly(L-lysine) (MW 5000) using 3,3′-dithiodipropionic acid as a disulfide linker between drug and polymer and succinic acid as a control linker. Conjugation occurred via esterification of a hydroxyl of DEX with the carboxyl of dithiodipropionic/succinic acid grafted to the polypeptide polymer. The average conjugate numbers of DEX were about 5.0 and 3.2 per polypeptide, and the drug loading was 17.1 and 13.2% for the dithiodipropionic- and succinic-based conjugates, respectively. Due to their amphiphilic nature, the synthesized conjugates self-assembled in PBS into micelles, with sizes of 66 and 76 nm, and ζ-potentials of −5.2 and −7.8 mV for the dithiodipropionic- and succinic-based conjugates, respectively. In vitro experiments showed that the DEX release from the dithiopropionic-linker based conjugate was higher at pH 5.0 than at pH 7.4 and was 49% at 120 h; 66% of the DEX was released at pH 7.4 when 10 mM glutathione was used as a trigger for destruction of the disulfide bonds. The DEX release from the succinic-based conjugates was slower (10% in 120 h) and insensitive to pH or glutathione. In vivo studies on a mouse colorectal cancer model showed that the intravenous administration of a redox and pH double sensitive conjugate dramatically increased the accumulation of DEX in the tumor when compared to free DEX. In addition, at an equal dose (10 mg/kg), this conjugate demonstrated excellent anti-tumor activity compared to free DEX: tumor suppression was 86 versus 49%, respectively.

Chaikomon et al. [137] synthesized the DEX-doxorubicin (DOX) conjugates to overcome the resistance of cancer cells that have the ability to actively remove DOX from the nucleus. The conjugate was derived via simple conjugation of the 3′amino group of DOX to the 3C atom of the DEX molecule via a linker of 2-iminothiolane. The conjugated product maintained cytotoxicity in MCF-7 cells (breast cancer cells) overexpressing multidrug resistance-1 that showed an approximately 16-fold higher resistance to DOX than wildtype cells.

Sau et Banerjee [138] developed newer cancer targeted molecules by conjugation of cationic lipid to DEX. For this, a cationic twin-carbon chain lipid is chemically conjugated with DEX. DEX derivatives differing in the length of the carbon chain linked to the quaternary nitrogen atom were first synthesized. The amino group of tert-butyl *N*-(2-aminoethyl)carbamate was reacted with alkyl bromides of different carbon chain lengths to obtain respective tertiary amines, and this was followed by *N*-deprotection to obtain the amines. Separately, DEX was oxidized using NaIO_4_ to produce a compound with a free carboxylic acid functionality that was subsequently coupled to the free amine using EDC/HOBT/DMAP to produce amide products. The resulting tertiary amines were quaternized with methyl iodide. An in vivo study in a highly aggressive B16F10-based murine melanoma model in C57BL6/J black mice demonstrated that this conjugate had potent antiangiogenic properties, including the selective JAK3/STAT3-reducing anticancer characteristics of a new DEX-based anti-tumor molecule.

Wang et al. [139,140] fabricated a targeted gene delivery system based on DEX-conjugated solid lipid nanoparticles aimed at increasing the nuclear uptake of genetic materials by the nuclear localization signal. DEX-21-mesylate was coupled to 6-lauroxyhexyl ornithinate (LHON) using 2-iminothiolane (Traut’s reagent). The in vivo transfection efficiency was 30% higher for the DEX-modified vectors than for the vectors not containing DEX in the model of disseminated peritoneal tumors in mice bearing KB cells.

Howard et al. [74] conjugated DEX to PEG-poly(aspartate) block copolymers (MW 12,000) using hydrazone, ester, or hydrazone-ester dual linkers to achieve pH-controlled drug release. Ketonic acids containing 3, 4, and 5 methylene groups (4-acetylbutyric acid [ABA], 6-oxoheptanoic acid [OHA], and 7-oxooctanoic acid [OOA]) were used as spacers to separate the dual linkers. DEX was conjugated via an ester or with hydrazone-ester (hydrazone-ABA-ester, hydrazone-OHA-ester, and hydrazone-OOA-ester) dual linkers by an esterification reaction between the C21 hydroxyl group of DEX and carboxyl groups of the copolymers. DEX loading was about 4.5–9%. The ester linker demonstrated an accelerated release of DEX at pH 7.4 and a suppressed release at pH 5.0. The hydrazone-ester dual linkers showed the opposite drug release patterns, with an accelerated release at pH5.0 and suppressed release at pH 7.4. Thus, conjugates with hydrazone-ester dual linkers would be expected to be stable in blood and to release DEX in the acidic environment of the tumor. In addition, DEX release was reduced at pH 5.0 as the chain length of the spacer increased but was not dependent on the spacer length at pH 7.4.

Kostkova et al. [141] synthesized HPMA copolymer-drug conjugates with DEX as an anti-inflammatory and anti-proliferative drug, and with the anticancer drug DOX. For the DEX-containing conjugates, the DEX was esterified either with levulinic acid (LEV) or with 4-(2-oxopropyl)benzoic acid using DCC. The esters were then attached to the polymer carrier via a spacer containing a 6-aminohexanoic acid residue and a hydrazone bond. DOX was attached to the same polymer carriers already bearing the DEX esters, also via a hydrazone bond. The resulting conjugates were 8–12 nm in size, and the loading efficiency was 2.3–4.8% and 6.6–9.9% for DEX and DOX, respectively. The conjugates were stable in solution at pH 7.4 (blood stream model), releasing 25% of the DEX and 4% of the DOX in 8 h. By contrast, about 90% of both DEX and DOX were released at pH 5 (endosomal pH) in 24 h. Both the in vitro and in vivo anticancer activity in mouse B-cell (38C13) and T-cell (EL4) lymphoma showed a synergistic effect (5–7 fold) of the two conjugated drugs, when compared to the treatments with a single drug conjugate or the free drugs.

Krakovicova et al. [142] synthesized a conjugate in which DOX and DEX were covalently attached to the copolymer of HPMA. Both drugs were attached to the polymer carrier via spacers containing pH-labile hydrazone bond. DEX esters were prepared by esterification of the C21 hydroxyl group with two keto acids, LEV or 4-(2-oxopropyl)benzoic acid. Both esters of DEX were attached to the polymer carrier via a hydrazone bond to a spacer containing a 6-aminohexanoic acid residue. DOX was attached to the same polymer carrier through the hydrazone bond formed in the condensation of its C13 carbonyl group with the hydrazide group of the polymer. The in vitro release of DEX and its ester (DEX-LEV) from the polymer conjugate showed a much lower release at pH 7.4 (30% of the sum of DEX and DEX-LEV in 2 h) than at pH 5 (more than 80% in 10 min).

### 4.5. Antigen-Drug Conjugates

The treatment of patients (e.g., those with multiple sclerosis) with immunomodulatory drugs including DEX is non-specific and can lead to global immunosuppression. Targeted delivery of antigen-GC conjugates to diseased cell populations can significantly reduce the level of this severe side reaction [89].

Pickens et al. [89] formed DEX-antigen conjugates for the treatment of multiple sclerosis using Cu(II)-catalyzed azide-alkyne cycloaddition with the inclusion of a hydrolyzable linker to maintain the activity of the released DEX. The proteolipid protein of the myelin sheath was used as an antigen; and 4-pentynoic acid was used as an ester linker capable of releasing DEX in the acidic environment. An azidoacetic acid NHS ester was used for N_3_-functionalization of the OH-group of DEX. A study of the release kinetics showed that DEX was released in 100 h in unbuffered solutions (pH 7.0) and was released completely in approximately 50 h at pH 5.5. At the same time, complete hydrolysis of the ester linkers occurred in a few hours, indicating that phosphate anions catalyze the effect via the nucleophilic attack on the ester bond. Subcutaneous administration of these DEX-antigen conjugates to mice induced with autoimmune encephalomyelitis protected them against the disease onset for 25 days, demonstrating enhanced efficacy compared with free DEX.

### 4.6. Gene Delivery Systems

The three crucial steps for efficient gene delivery are cellular uptake, endosomal release, and nuclear translocation [143]. Nuclear transportation is one of the main barriers to overcome when choosing which adducts are used, as these can act as signals of nuclear localization. For example, DEX has intracellular receptors and can efficiently enter the nucleus through a steroid-mediated gene delivery mechanism [98,144,145,146,147].

Jeong et al. [79] designed DEX-polyethylenimine (PEI) conjugates complexed with a minicircle plasmid (hydrodynamic size of approximately 200 nm) as a gene delivery system to adipose-derived stem cells for induction of synergistic effects with chondrogenic genes. DEX was covalently conjugated to PEI using carbodiimide chemistry. In vivo experiments using surgically-induced osteoarthritis showed that conjugate-treated rats had significantly lower levels of COX-2 (4–5 times) and matrix metalloproteinase-13 (5–10 times) in synovial fluids, as well as significantly less joint destruction, than were observed in control rats.

Malaekeh-Nikouei et al. [99] developed gene carriers via conjugation of DEX (5, 10, and 20%) to the polypropylenimines (PPI) of 4 and 5 generations (cationic dendrimers with terminal amino groups, potent non-viral vectors) the 21-OH group of DEX was first modified with methanesulfonyl chloride to activate it, and it was then conjugated to PPI using Traut’s reagent (2-iminothiolane; a thiolating reagent that reacts with primary amine groups to form sulfhydryl groups). The DEX degrees of substitution were 4, 8, and 15%. The resulting particles had a size of 170–400 nm, a ζ-potential that varied from +20 to +30 mV. The addition of DEX increased the transfection by 1.3–1.6 times and reduced cytotoxicity by 2–5 times.

Malaekeh-Nikouei et al. [98] obtained DEX-conjugates with PEI (MW 10,000) via the 2-iminothiolane method. The resulting conjugates had a size of 130–500 nm and a ζ-potential of −10 to + 45 mV; they were used to form lipopolyplexes for plasmid delivery and this improved transfection activity by up to 2.8 times, compared to similar free DEX systems. Malaekeh-Nikouei et al. [97] also synthesized conjugates of DEX with polyallylamines (MW 15,000 and 65,000) by the 2-iminothiolane chemistry for nuclear delivery of plasmid DNA. The DEX degree of substitution varied from 2.7 to 9.8%. The resulting polyplexes had a size of 145–700 nm, and a ζ-potential of −23 to +25 mV. The polyallylamine (MW 15,000)-based polyplexes showed better cell transfection of the Neuro2A cell line than those based on polyallylamine (MW 65,000). In total, the modification with DEX significantly reduced the cytotoxicity and slightly decreased transfection compared to the free polyallylamine.

Choi et al. [96] developed a multi-target delivery system for treating asthma by combining DEX with a small interfering RNA (siRNA). The complex was formed by first obtaining a conjugate of DEX-21-mesylate with PEI (MW 2000) (PEI2k) via a linker of 2-iminothiolane (Traut’s reagent) and then adding Vitamin D binding protein and siRNA. The resulting particles had a size of about 400 nm and a ζ-potential of about +47 mV. An in vivo study on a mouse model showed that the developed conjugates effectively reduced airway inflammation, goblet cell hyperplasia, and expression of IL-4, IL-13, and CCL11.

Choi et al. [148] also used the complex of DEX-PEI2k with siRNA for treatment of acute lung inflammation. In vivo studies in a SiO_2_-treated Beas-2b cells model and in a BALB/c mouse model with SiO_2_-induced acute lung inflammation showed a better limitation of the pneumonia reaction in cells and mice by the DEX conjugates than by DEX-free complexes. This demonstrated the potential of DEX as a carrier for siRNA delivery to the nucleus.

Kim et al. [92,95] developed a DEX-PEI2k conjugate as a gene carrier for plasmid delivery into glioblastoma cells. For this, DEX-21-mesylate was conjugated with PEI by a one-step reaction using Traut’s reagent (1 mole of DEX was conjugated per 1.3 mol of PEI). An in vivo study in subcutaneous and intracranial glioblastoma models showed that the tumor size was reduced more effectively when using DEX-based carriers than in the control group.

Kim et al. [92,94] designed conjugates of DEX with low MW PEI2k for plasmid DNA (pDNA) delivery. Briefly, DEX-21-mesylate was coupled with PEI using Traut’s reagent. An in vitro transfection study of L2 lung epithelial cells revealed a higher (about 10 times) transfection efficiency for the DEX conjugates than for PEI or for a simple mixture of PEI and DEX. The in vivo anti-inflammatory effect of DEX-conjugate in a BALB/c mice model of lipopolysaccharide induced acute lung injury was higher than that of controls: IL-6 level and TNF-α level decreased 3 and 4 times, respectively.

Kim et al. [149] conjugated DEX to linear PEI (MW 25,000, PEI25k) using Traut’s reagent method [150] to improve the efficiency of gene delivery. The amount of conjugated DEX was 23 DEX units per 1 molecule of PEI25k. The resulting conjugate had higher transfection efficiency than PEI25k (~4.5 times) and lipofectamine 2000 (~40 times).

Kim et al. [92,93] also synthesized conjugates of DEX with low MW PEI2k to use the anti-apoptotic effect of DEX in ischemic disease gene therapy. A transfection assay into H9C2 rat cardiomyocytes showed higher transfection efficiency (1.5 times) and lower cytotoxicity (1.2 times) for DEX-PEI2k than for PEI25k.

Bae et al. [92] synthesized DEX conjugates with low MW PEI2k using Traut’s coupling reagent for improved efficiency of DNA translocation into the nucleus. The resulting conjugate contained 1 mole of PEI2k per 1.3 mol of PEI. An in vitro transfection assay conducted on HEK293 cells and HepG2 cells showed a higher gene delivery efficiency (5–20 times) for the DEX-PEI2k/DNA complex than the PEI2k/DNA complex.

Young Kim et al. [151] designed conjugates of DEX with polyamidoamine (PAM) dendrimers of generation 1 (PAMG1) and PAM generation 2 (PAMG2) to increase their transfection efficiency. DEX was conjugated with PAMG1 and PAMG2 using Traut’s chemistry. The molar ratios between the conjugated DEX and the PAM were 0.3:1 and 2.4:1 for PAMG1 and PAMG2, respectively. The developed conjugates had higher transfection efficiencies (8–10 times) than PAMG1, PAMG2, PEI25k, and lipofectamine (in HEK293 cells and N2A cells), and were less cytotoxic, as determined by MTT (3-(4,5-dimethylthiazol-2-yl)-2,5-diphenyl tetrazolium bromide) assay, in HEK293 cells than lipofectamine (1.4 times). In addition, the conjugates decreased the TNF-α level more efficiently (1.4 and 4.7 times) than free DEX in lipopolysaccharide (LPS)-induced Raw264.7 cells.

Wang et al. [140] synthesized DEX-conjugated lipid as the material of solid lipid nanoparticles to increase the nuclear uptake of genetic materials for targeting cells through receptor-mediated pathways and the nuclear localization signal. For this, DEX was conjugated to LHON [150]. Briefly, DEX coupling to LHON was performed with Traut’s reagent and DEX-21-mesylate; as a result, 2 mol of DEX was conjugated per 1.2 mol of LHON. The synthesized DEX conjugate was used to obtain a complex with a transferrin-PEG-phosphatidylethanolamine ligand. Both in vitro (in human hepatoma carcinoma cell lines, HepG2 cells) and in vivo (against HepG2 solid tumors in mice model) tests confirmed that the transfection efficiency of the DEX modified vectors was 1.5–2 times higher than DEX free carriers.

Choi et al. [148] synthesized a conjugate of DEX with PAMAMG4 as a gene carrier for efficient nuclear translocation by a previous method [22], with some modifications. The transfection efficiency was 10 times higher for the obtained conjugates than for PAMAM or PEI. Confocal microscopy studies also showed that the DEX conjugate/DNA complexes were localized mainly in the nucleus.

Jeon et al. [152] developed DEX-conjugated PAMAM generation 1 (PAMAMG1) and generation 2 (PAMAMG2) for brain delivery of the heme oxygenase-1 gene for the treatment of ischemic stroke. Conjugates were synthesized by a cross-linking reaction using Traut’s reagent and DEX-21-mesylate. The molar ratio between the conjugated DEX and the PAMAM was 2.4:1. The obtained complexes had a particle size of approximately 170 nm and a positive ζ-potential of approximately 56 mV. The pDNA delivery efficiency into Neuro2A cells was about 1.5 times higher for the developed conjugates than for PEI25k, DEX-conjugated PEI, or free PAMAM.

Hyun et al. [128] developed a delivery system based on heme oxygenase-1 gene and DEX by conjugation of DEX to PEI2k for combinational therapy of the ischemic brain. DEX-PEI2k was synthesized using Traut’s reagent and DEX-21-mesylate [92]. The DEX-PEI2k conjugates had a higher transfection efficiency than the simple mixtures of DEX and PEI2k (20 times), PEI2k (10 times), or lipofectamine (5 times). The MTT assay indicated that the DEX-PEI2k conjugate was 1.4 times less toxic to cells than lipofectamine.

## 5. Conclusions

DEX conjugates are very widely used: first, as DEX delivery systems to improve GC pharmacological effects (anti-inflammatory, immunomodulatory, etc.); second, as anti-tumor delivery systems that including combinations with chemotherapeutic drugs; and third, as gene delivery systems.

The rate of DEX release from conjugates depends on the molecular weight of the polymers, the synthesis method (RAFT polymerization can result in systems with zero order release kinetics, and are characterized by the most sustained release), and the type of linker (hydrazone linkers provide the best sustained release). The prolongation of DEX action reduces the dose and frequency requirements for administration of the drug. In addition, the type of linker ensures the stability of the conjugate under physiological conditions and its hydrolysis with the release of active DEX in pathological environments (e.g., inflammation sites and tumors). For example, anti-tumor delivery systems often use *S-S*-linkers (cysteine, dithiopropionic acid, etc.). This approach allows protection of healthy organs and tissues and ensures targeted delivery of DEX to the pathological site, while increasing the bioavailability and reducing toxicity and side effects.

Most often, DEX delivery systems are used to treat inflammatory joint diseases. However, these systems also have great therapeutic potential for intravitreal delivery. Targeted delivery of DEX-conjugates into the vitreous humor of the eye will significantly reduce the drug dose and frequency of side effects, and the prolonged release will reduce the number of required injections. In addition, a small number of studies are focused on conjugates of DEX with natural polysaccharides, which are non-toxic and biocompatible macromolecules suitable for simple carbodiimide synthesis techniques. These types of conjugates is of interest for medical application, including intravitreal delivery of DEX. In this context, the delivery systems based on HA, which is a natural component of the vitreous humor, have great potential.
